# Therapeutic Notes

**Published:** 1901-04-27

**Authors:** 


### THERAPEUTIC NOTES.

### PERSULPHATE OF SODA.

Persulphate of soda, or persodine, as it has also been called, is a powerful oxidising agent, has antiseptic properties, and is to some extent antithermic. It has been employed in Germany and in France in tuberculosis, and in convalescence from acute diseases, or other affections attended with debility.

Amongst those who have made a study of its properties are R. Friedlander,[1] Joseph Nicolas, of Lyons,[2] and M. J. Garel, of Lyons.[3] It is a tonic, and active stimulant of the appetite, and eupeptic, and is feebly toxic, requiring doses of 6 grains and upwards per kilogramme of weight to have a fatal effect on animals.

It is recommended to be given fasting about 1½ hour before the first meal, in the dose of 4 to 6 grains for adults, and in two days we may expect the appetite to increase markedly, and frequently to a striking extent. Slight temporary diarrhœa is sometimes observed, but no other inconvenience. It has not yet been tried sufficiently extensively to establish its proper place in therapeutics, it but is well worth trial.

[1] Fried. Ther. Mon., Feb. 1899. [2] Nicol. Com. Soc. de Biol. Prov. Med., April 28, 1900, and following Nos. [3] Garel. Bull. Méd., Aug. 22, 1900.

