# Persulphate of Soda

**Published:** 1901-05-11

**Authors:** 


### A CORRECTION.

A Correspondent draws our attention to an error in a note on Persulphate of Soda on page 67, in which "grains" is a misprint for "grammes."

